# Budget impact analysis of implementing point-of-care molecular testing for influenza A&B and RSV in Saudi older adult patients across hospital and home-care settings

**DOI:** 10.1186/s13561-026-00872-3

**Published:** 2026-10-07

**Authors:** Walid Alkeridy, Khulud Alharbi, Amal Abuhamar, Abdullah Alarifi, Najim Z. Alshaharni, Sulafa Alrkabi, Gihan Hamdy Elsisi, Saad M. Alsaad

**Affiliations:** 1 Department of Medicine, College of Medicine, King Saud University, Riyadh, Saudi Arabia; 2 Department of Medicine, Division of Geriatric Medicine, University of British Columbia, Vancouver, BC Canada; 3 Therapeutic Affairs Deputyship, General Administration of Home Health Care, Ministry of Health, Riyadh, Saudi Arabia; 4 Department of Health Administration and Hospitals, College of Public Health and Health Informatics, Umm Al-Qura University, Makkah, Saudi Arabia; 5 Ministry of Health, Riyadh, Saudi Arabia; 6 Deputy of Population Health, Ministry of Health, Riyadh, Saudi Arabia; 7 Department of Family & Community Medicine, Faculty of Medicine, University of Jeddah, Jeddah, Saudi Arabia; 8 Faculty of Health & Medicine, Lancaster University, Lancaster, UK; 9 Managing Director, HTA Office (Technology Appraisal), Middle East and North Africa, Cairo, Egypt; 10 Department of Family and Community Medicine, College of Medicine, King Saud University, P.O. Box 11495, Riyadh, Saudi Arabia; 11 College of Medicine, Prince Faisal Bin Bandar Scientific Chair for Geriatric Research, King Saud University, Riyadh, Saudi Arabia; 12 American University in Cairo, Cairo, Egypt; 13 International Society for Pharmacoeconomics and Outcomes Research (ISPOR), Cairo, Egypt; 14 World Health Organization EMR Office & USAID, Cairo, Egypt

## Abstract

**Introduction:**

The aim of our study is to compare the economic impact of adopting the molecular point-of-care test (mPOCT) for influenza A&B and RSV with current clinical judgment in Saudi Arabia, in both hospital-based and home care older adults.

**Methodology:**

A static Budget Impact Analysis (BIA) was developed in Microsoft Excel to evaluate the clinical and economic consequences of adopting mPOCT for influenza A&B and RSV testing among prevalent older adult patients in Saudi Arabia. The model adopted the perspective of the Saudi healthcare system over a one-season horizon, comparing two diagnostic strategies: current practice based on clinical judgement and a future scenario using mPOCT in both older-adult-friendly hospitals and homecare settings. Parallel decision-tree structures were constructed for influenza and RSV, incorporating test accuracy, diagnostic outcomes, treatment pathways, hospitalization requirements, and mortality. Costs included diagnostics, staff time, outpatient visits, hospital stays, medications, and resources used for complications and inappropriate antibiotic prescribing. Epidemiologic, clinical, and cost inputs were derived from published evidence and supplemented with Saudi-specific data where available. A deterministic sensitivity analysis varied cost parameters ± 20% and clinical parameters across published confidence intervals.

**Results:**

Adoption of the mPOCT was associated with substantial cost savings across all modeled (hospital and homecare settings) older adults. For Influenza A&B, total costs decreased from SAR 57,858,038 to SAR 26,933,837 in hospital settings and from SAR 13,254,535 to SAR 7,974,232 in homecare, yielding savings of SAR 30,924,201 and SAR 5,280,303, respectively. For RSV, hospital costs fell from SAR 40,640,192 to SAR 20,706,660, and homecare costs from SAR 9,157,462 to SAR 5,988,728, saving SAR 19,933,531 and SAR 3,168,733, respectively. Overall, mPOCT produced SAR 59,306,768 in combined savings, demonstrating strong economic value for respiratory infection management in Saudi Arabia.

**Conclusion:**

This BIA demonstrates that implementing the mPOCT platform for influenza A&B and RSV in Saudi Arabia may provide substantial clinical and economic value, particularly for high-risk older adults. By enabling rapid and accurate diagnosis at both hospital and homecare entry points, mPOCT may significantly reduce false negatives, mitigates progression to severe lower respiratory tract infections, and decreases unnecessary antiviral and antibiotic use. These improvements could translate into major reductions in healthcare utilization, including ward and ICU admissions, and may generate considerable cost savings across a single respiratory season.

**Supplementary Information:**

The online version contains supplementary material available at https://doi.org/10.1186/s13561-026-00872-3.

## Introduction

Influenza is a contagious respiratory infection caused by influenza viruses that target the nose, throat, and, in some cases, the lungs [1]. Seasonal influenza is primarily driven by human influenza A and B viruses, both of which undergo continuous genetic changes that can reduce pre-existing immunity and increase susceptibility within the population [2]. The most typical symptoms include cough and fever [3], and while many infections remain mild, severe illnesses can occur. Influenza spreads mainly through respiratory transmission from person to person, and infected individuals may remain contagious for up to 7 days [4]. The true incidence of influenza is difficult to measure because many infected individuals do not seek medical care; however, estimates that include asymptomatic infections suggest that 3.5% to 15.2% of the global population is infected each season [5]. Data from 33 countries, representing more than half of the world’s population, indicate that influenza leads to 291,243–645,832 respiratory deaths annually, corresponding to 4.0–8.8 deaths per 100,000 individuals [6]. Hospitalizations represent another major component of the influenza burden, with the United States reporting 100,000 to 710,000 influenza-associated hospitalizations each year since 2010 [7, 8]. A proportion of these patients require intensive care, with infants facing the greatest likelihood of intensive care unit (ICU) admission [9]. Even mild influenza cases have economic consequences, including absenteeism from work or caregiving responsibilities [10, 11].

Influenza can also lead to significant medical complications, including pneumonia [12, 13], bacterial superinfection [14], myocarditis, encephalitis, myositis, rhabdomyolysis, and multiorgan failure [15]. Certain groups, including adults aged 65 years and older [12, 16–20], young children under five years [21–24], and individuals with chronic conditions such as asthma or diabetes [12, 16, 25, 26], face elevated risks of severe outcomes. Coinfections with SARS-CoV-2 are increasingly common [27–30] and are associated with worse clinical outcomes than infection with either virus alone [28, 31, 32]. The economic burden of influenza is substantial in the United States [33] and in Europe [34]. Vaccination remains the most effective preventive strategy for influenza [35–38], though vaccine uptake varies across settings [39–41] and effectiveness fluctuates by season [42]. Diagnostic approaches include multiple test types, with guidelines recommending rapid molecular assays for individuals at the highest risk [35, 43]. Nucleic acid amplification tests (NAATs) are considered the diagnostic gold standard due to high sensitivity and specificity, and rapid tests have increasingly enabled timely clinical decisions [35]. Antiviral therapy is recommended for hospitalized patients, those with severe or progressive illness, and high-risk groups [35], and is most effective when initiated within the first 48 h of symptom onset [44]. However, despite available vaccines and antiviral agents, influenza continues to impose a considerable burden, and there remains a need for broader use of accurate mPOCT [45, 46]. Wider adoption of rapid, highly sensitive mPOCT could improve clinical management, optimize resource use, and reduce transmission by supporting appropriate isolation measures [47–49]. Molecular POCT further enables rapid, accurate diagnosis and has the potential to significantly reduce the burden of influenza-associated disease [45, 50–55].

The ID NOW™ Influenza A & B2 assay provides rapid molecular detection and differentiation of influenza A and B viral RNA [56]. This assay is one of the fastest molecular point-of-care platforms available [57, 58], offering workflows that integrate efficiently into routine clinical settings [52, 59, 60]. The test is supported by substantial clinical evidence indicating high sensitivity and specificity, low invalid rates [57, 60–64], reductions in unnecessary antibiotic prescribing [51, 53, 61], and increased appropriate antiviral use [51, 65].

Respiratory syncytial virus (RSV) is another highly prevalent and contagious virus responsible for a wide range of respiratory illnesses [66]. It is a medium-sized, negative-sense, single-stranded RNA virus within the Pneumonia virus genus and shares phylogenetic similarities with measles, mumps, and parainfluenza viruses [66, 67]. Symptoms range from mild upper respiratory infection to life-threatening lower respiratory disease [66, 68]. Older adults and high-risk patients experience high RSV-associated hospitalization rates [69–71]. Global estimates indicate that RSV affects approximately 64 million people annually, though available data remain limited [72]. RSV accounts for an estimated 199,000 child deaths annually worldwide, with 99% occurring in low- and middle-income countries [66, 73]. RSV is believed to cause 11,000–17,000 deaths annually in US older adults and around 8,000 deaths each year in the UK [71, 74].

Hospitalization is a key component of the RSV burden in older adults [75]. Pneumonia occurs in 44% of hospitalized older adult RSV patients [76], and cardiovascular complications, including heart failure and arrhythmias, occur in 14–22% of cases [77]. RSV coinfection with other respiratory viruses, including COVID-19 or influenza, can worsen disease severity and prolong hospital stays, increasing treatment requirements and impairing immune responses [78, 79]. The clinical burden of RSV contributes to substantial direct costs (e.g., treatment, hospitalization) and indirect costs (e.g., time off work for illness or caregiving) [80, 81].

Routine pathogen identification is uncommon in mild respiratory illness, but the CDC recommends RSV testing for high-risk individuals during periods when influenza and SARS-CoV-2 are co-circulating [82]. Treatment of RSV remains primarily supportive [83]. Diagnostic testing is valuable for differentiating RSV from other viral illnesses, as clinical presentation alone is often nonspecific [84]. Wider adoption of accurate mPOCT could support testing-based clinical management strategies, including implementation of isolation measures to reduce transmission [68], and more appropriate antibiotic use, helping mitigate antimicrobial resistance [85, 86]. Rapid mPOCT may also decrease overall resource use, including emergency department throughput times [47, 50, 87].

The ID NOW™ RSV assay uses isothermal nucleic acid amplification for rapid, qualitative detection of RSV RNA [88]. Clinical studies demonstrate strong performance compared with laboratory RT-PCR [89–91], with comparable sensitivity for both direct swabs and swabs in transport medium [91]. Evidence from influenza testing also shows reductions in unnecessary antibiotic use [51, 55, 61], emergency department length of stay [51, 52, 55], hospital resource use [52, 53], and healthcare costs [65, 92]. The mPOCT for RSV similarly improves resource utilization and reduces associated expenditures [50, 93].

In Saudi Arabia, care for older adults is integrated across public and private providers, with the Ministry of Health assuming a pivotal role in the country’s healthcare system, driving national objectives for healthy ageing, quality of care, service accessibility, and reducing morbidity and mortality from high-burden conditions. By advancing programs in home and hospital-based services, the Ministry of Health continues to strengthen the care continuum for older adults.

The Age-Friendly Health Facilities Initiative, a national project initiated in 2023, stands as a testament to the Ministry’s commitment to elevating the standards of quality, safety, and clinical excellence for older adults across the Kingdom. This is achieved through the formal accreditation of healthcare facilities in accordance with age-friendly standards. The initiative commenced with the accreditation of four hospitals in 2023 and significantly scaled to 43 accredited facilities by October 2025. A core pillar of the Age-Friendly Health Facilities Initiative is the prioritization of preventive care with a specific focus on safeguarding older adults, particularly through streamlined vaccination programs against influenza, RSV, and herpes zoster, while establishing a more conducive framework for early diagnosis and prompt management, with the intent of improving outcomes for lower respiratory tract infections in this vulnerable demographic.

Rapid antigen tests are rarely used in clinical practice and remain limited to select hospitals, leading to inappropriate antibiotic use, delayed antiviral treatment, and prolonged emergency department congestion, therefore leaving significant gaps in routine care for high-risk groups, especially older adults [94]. These challenges in Saudi Arabia underscore the need for widespread implementation of rapid mPOCT for influenza and RSV within the Saudi healthcare system. In addition, early differentiation between influenza, RSV, and other respiratory pathogens facilitates targeted therapy, reduces isolation bed occupancy, and frees hospital resources for more critical cases.

## Objective

The objective of this study is to evaluate the clinical and economic impact of implementing the mPOCT for the rapid diagnosis of influenza A&B and RSV, compared with current practice based on clinical judgement alone, within the Saudi healthcare system for both older adults’ homecare and hospital-based populations.

This evaluation is particularly valuable because clinical judgement alone often leads to diagnostic uncertainty due to overlapping symptoms among viral respiratory infections, resulting in delayed or inappropriate treatment, unnecessary laboratory testing, and increased healthcare costs.

## Methodology

### Study design

Our budget impact model was developed from the perspective of the Saudi healthcare system, incorporating both older adults’ homecare and hospital-based populations to reflect real-world clinical pathways and resource utilization in Saudi Arabia, aligning with International Society for Health Economics and Outcomes Research (ISPOR) budget impact analysis good practice. The model structure was designed to evaluate the clinical and economic consequences of adopting the mPOCT for the rapid diagnosis of influenza A&B and RSV, compared with the current practice of clinical judgement alone.

To inform this economic evaluation, a targeted literature review was conducted to extract relevant data on diagnostic accuracy, time to treatment, healthcare resource use, and downstream clinical outcomes associated with mPOCT versus symptom-based diagnosis.

Because local data reflecting Saudi clinical practice in respiratory infection management are limited, both pre-implementation and post-implementation structured interviews were conducted with 4 Saudi clinicians from homecare older adults services and older adults hospital care settings (Makkah Health Cluster, Yanbu General Hospital at Madinah Health Cluster, Buraydah Central Hospital at Qassim Health Cluster, West Home Healthcare center at Madinah Health Cluster) and 4 healthcare decision-makers from Ministry of Health. The pre-implementation interviews aimed to document current diagnostic pathways based on clinical judgement, resource utilization patterns, and operational challenges in managing influenza and RSV. Following the introduction of mPOCT, post-implementation interviews were carried out to assess changes in workflow efficiency, diagnostic accuracy, treatment decisions, and overall system performance. Eight interviews followed a structured, pre-designed questionnaire (supplementary index) to validate key model assumptions, compare real-world clinical practice before and after mPOCT adoption, and ensure that the model accurately captures Saudi-specific practice patterns and consensus was reached over three rounds using a quasi-Delphi approach. These insights were essential for contextualizing model parameters and aligning cost estimates with actual clinical workflows in Saudi facilities, particularly for the older adult Saudi population during peak respiratory seasons. Four clinicians were interviewed in their professional capacity as professionals and four healthcare decision-makers, and no patient-level data or personal identifiers were collected. The main researcher informed the Delphi panel about the research purpose, anonymity provisions, and data storage methods used. The experts were fully informed of research aims, and their right to confidentiality. The main researcher informed the Delphi panel about the research purpose, anonymity provisions, and data storage methods used. The experts were fully informed of research aims, and their right to confidentiality.

### Model design

A static budget impact analysis (BIA) model was developed in Microsoft Excel© to evaluate the clinical and economic implications of introducing the mPOCT platform for the rapid diagnosis of influenza A&B and RSV among Saudi older adult patients presenting with acute respiratory illness. Given the acute, self-limited nature of influenza and RSV infections, a one-season (one-year) time horizon was applied to capture all direct medical costs attributable to a single respiratory episode.

The analysis compared two diagnostic strategies:

- Current scenario: respiratory infections are managed based primarily on clinical judgement.
- Future scenario: mPOCT is adopted as the frontline diagnostic tool for older adult patients with respiratory symptoms in homecare visits and hospital emergency departments, guiding immediate treatment decisions, isolation measures, and subsequent levels of care.

**Fig. 1:**
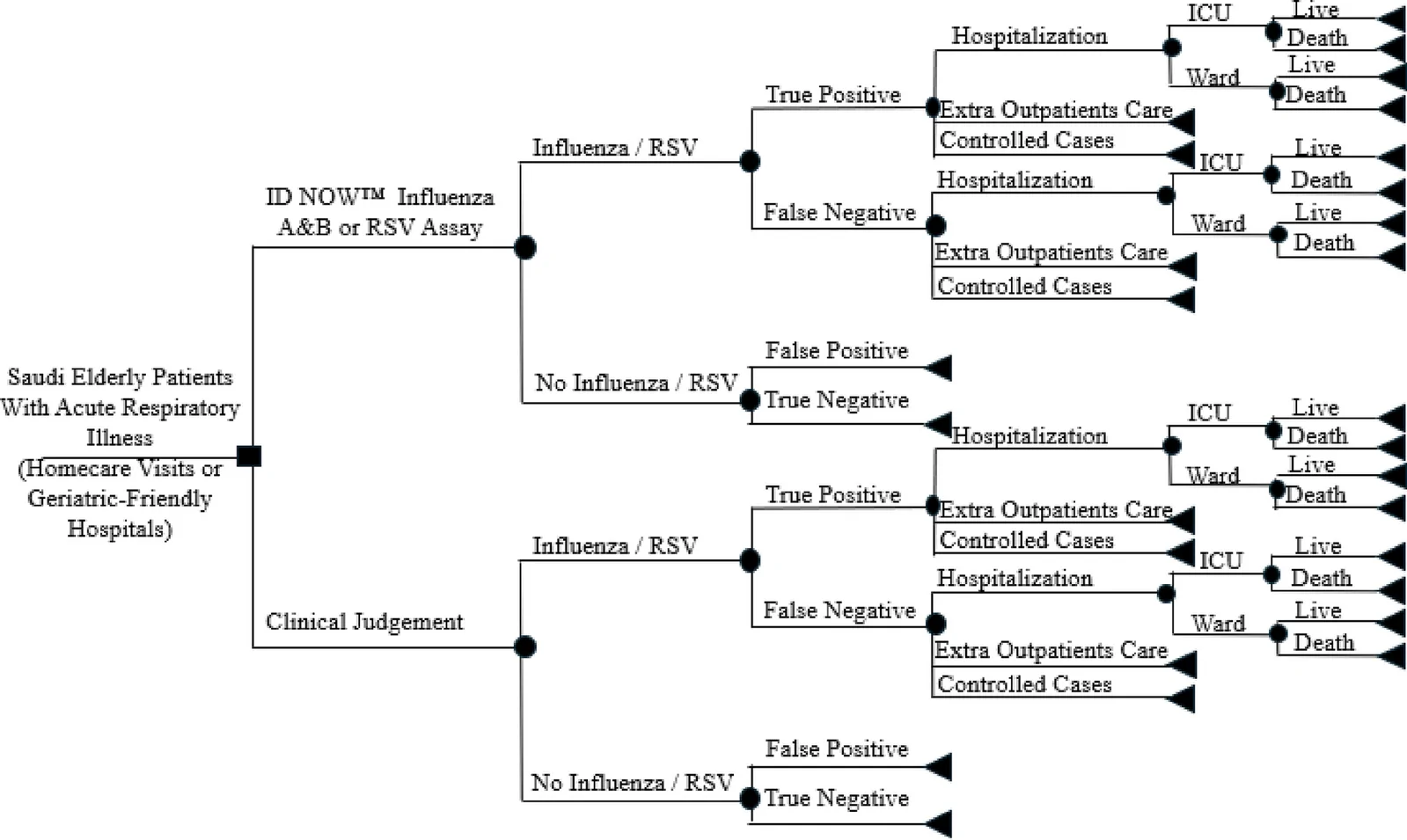
Decision tree structure for the diagnosis and management of influenza A&B/RSV in Saudi older adult patients using mPOCT versus clinical judgement. Where RSV: Respiratory Syncytial Virus

The decision tree (Fig. 1) begins with a hypothetical cohort of older adult Saudi patients presenting with respiratory illness—either in hospital or during a homecare visit—and then branches into two diagnostic strategies: mPOCT versus clinical judgement. Influenza A&B and RSV were modeled as independent pathways using the same decision tree structure, allowing each virus to retain its distinct epidemiology while enabling separate estimation of costs and clinical outcomes. Within each arm, patients are categorized as having influenza/RSV or no influenza/RSV, and are further classified as true positive, false negative, false positive, or true negative based on the diagnostic approach.

For those with influenza or RSV, downstream pathways include recovery managed in outpatient care, the need for additional outpatient visits, or hospitalization. Hospitalized patients may require ward care or admission to the ICU, with final health outcomes of survival or death. Mortality was modeled only among hospitalized patients, with probabilities varying by ward and ICU settings.

The model incorporates costs associated with mPOCT and conventional diagnostics, outpatient consultations, hospital ward and ICU stays, antibiotic and antiviral use, and additional resource utilization related to uncontrolled disease. For false-positive cases, which often lead to inappropriate antibiotic prescribing in older adult populations, antibiotic use was incorporated explicitly as an outcome influenced by diagnostic accuracy.

Epidemiologic inputs, diagnostic performance, and treatment patterns were informed by published literature (Table 2) and, where available, local Saudi data. Pre-implementation expert meetings with clinicians and decision-makers in older adult’s homecare and hospital settings were used to characterize current clinical judgment pathways and resource use, while post-implementation expert meetings following mPOCT adoption informed changes in workflow, treatment decisions, and utilization. These expert insights were essential for validating model assumptions and ensuring that parameter values reflect real-world Saudi practice.

The reductions in AMR-related costs were calculated by comparing the total AMR-related costs between the current clinical judgement scenario and the mPOCT scenario.

**Fig. 2:**
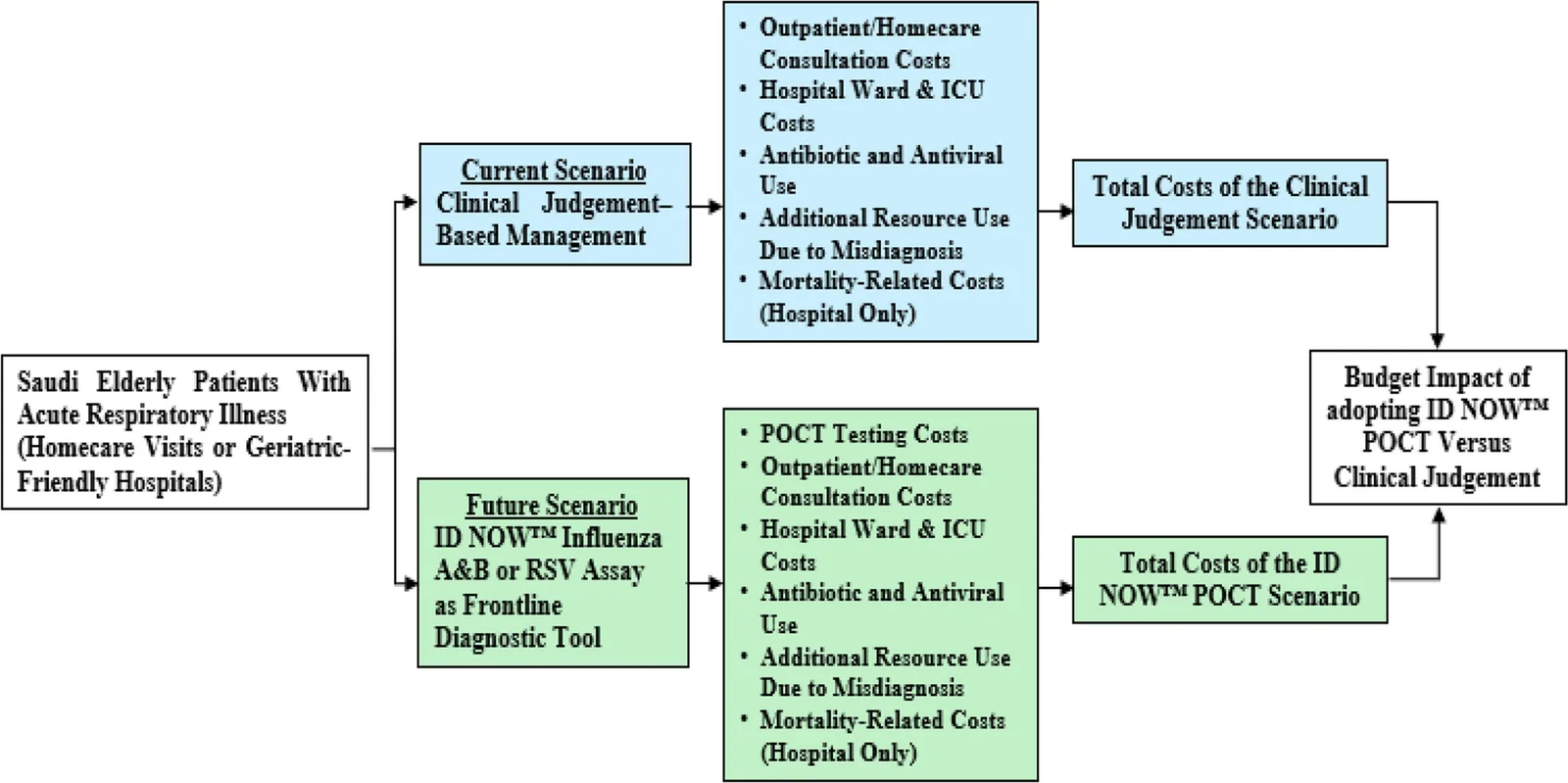
Overview of the budget impact analysis model evaluating mPOCT adoption for respiratory infection management in the Saudi older adults. Where RSV: Respiratory Syncytial Virus

The primary outcomes of the model are the incremental budget impact and the potential cost offsets associated with replacing clinical judgement alone with mPOCT in the management of respiratory infections in Saudi older adult populations. The model structure is illustrated in Fig. 2.

### Population

The BIA focuses on the prevalent older adult population in Saudi Arabia, a demographic characterized by an elevated risk of respiratory infections and higher healthcare utilization. Saudi Arabia has an estimated 35.3 million residents [100], of whom 5.5% (≈1.94 million) are aged 60 years and above [101]. Within this cohort, 58,445 individuals receive formal homecare services. Expert panel estimates that approximately 65% of these individuals are older adults, and all are included in the model population [102]. Furthermore, data from the MoH Statistical Yearbook and the General Authority for Statistics (GASTAT) indicate that 253,511 older adults receive hospital-based care (Table 1).

**Table 1:** Estimated population inputs for the budget impact analysis of respiratory infection mPOCT in older adults patients in Saudi Arabia

Population	Percentage	Number	Reference
Total Saudi Population receiving formal homecare services	–	58,445	[102]
Older adults home care Patients	65%	37,989	Expert panel
Older adults receive hospital-based care	–	253,511	MoH statistical year book and The General Authority for Statistics (GASTAT)
Influenza A&B in Older adult Patients			
Percent of Upper Respiratory Infection Seeking Medical consultation	20% of older adults home care and hospital based care	58,300	[103] – Assumed that all older adult Population seek medical consultation
% of Influenza A&B	19.2% of 58,300	11,194	[96]
Respiratory Syncytial Virus (RSV) in older adult Patients			
Incidence of Lower Respiratory Infection	15% of older adults home care and hospital based care	43,725	[95]
% of RSV	19% to 21% of 43,725	8,745	[94]

Epidemiological data indicate that 20% of older adults seek medical consultation for upper respiratory infections (URTI) [103], with 19.2% attributable to Influenza A & B [96]. Lower respiratory tract infections (LRTIs) affect roughly 15% of older adults annually [95], with 19–21% of these cases attributable to RSV [94]. These estimates form the basis for modeling disease burden and mPOCT adoption across homecare and hospital settings.

### Clinical inputs

All models’ clinical inputs are presented in Table 2. Clinical inputs influenza A and B and RSV were sourced from published diagnostic performance studies, epidemiological analyses, and hospital-based outcome research and validated from our Delphi panel. For both pathogens, the model incorporates test accuracy for mPOCT and for clinical judgment, enabling comparison between mPOCT and standard practice. The table details the probabilities of true and false diagnostic outcomes and the corresponding downstream consequences.

**Table 2:** Clinical inputs for the budget impact analysis decision tree

Clinical Inputs for Influenza A&B Decision Tree		
Number of Cases of Influenza		
Influenza A&B Cases	19.20%	[95]
No Influenza A&B Cases	80.80%	Derived
ID™ NOW Accuracy for Influenza A&B		
ID™ NOW—Both Influenza A&B Sensitivity	0.978	[60]
ID™ NOW—Both Influenza A&B Specificity	0.98	[60]
ID™ NOW Influenza A&B Probability False Negative	0.02	Derived
ID™ NOW Influenza A&B Probability False Positive	0.02	Derived
Clinical Judgement Accuracy of Influenza A&B		
Influenza A&B Clinical Judgement Sensitivity True Positive	0.29	[96]
Influenza A&B Clinical Judgement Specificity True Negative	0.92	[96]
Influenza A&B Clinical Judgement Probability False Negative	0.71	Derived
Influenza A&B Clinical Judgement Probability False Positive	0.08	Derived
Clinical Consequences of Not Initiating Antiviral Treatment for Influenza A&B		
Outcomes for influenza cases without antiviral initiation—Hospitalization	0.018	[96]
Outcomes for influenza cases without antiviral initiation—Extra Clinic Visit	0.478	[96]
Outcomes for influenza cases without antiviral initiation—Uncomplicated Cases	0.504	Derived
Clinical Consequences of Initiating Antiviral Treatment for Influenza A&B		
Odds ratio of extra outpatient visit with antiviral initiation	0.34	[96]
Odds ratio of hospitalization with antiviral initiation	0.35	[96]
Outcomes for influenza cases with rapid antiviral initiation—Hospitalization	0.0063	Derived
Outcomes for influenza cases with rapid antiviral initiation—Extra Clinic Visit	0.16252	Derived
Outcomes for influenza cases with rapid antiviral initiation—Uncomplicated Cases	0.83118	Derived
Influenza A&B ICU Hospitalization Clinical Inputs		
Hospitalization for influenza—ICU admission rate	0.105	[96]
ICU Length of Stay	8 Days	[96]
Mortality (with ICU admission)—Influenza	0.111	[96]
Living Cases (with ICU admission)—Influenza	0.889	Derived
Influenza A&B Ward Hospitalization Clinical Inputs		
Hospitalization for influenza—Ward admission rate	0.895	Derived
Ward Length of Stay	6 Days	[96]
Mortality (with Ward admission)—Influenza	0.052	[96]
Living Cases (with Ward admission)—Influenza	0.948	Derived
Influenza A&B Outpatients Clinical Inputs		
Duration of outpatient Influenza illness (untreated)	7 Days	[96]
Accelerated factor with early Antiviral Initiation	0.56	[96]
Duration of outpatient Influenza illness (Treated)	4 Days	Derived

Clinical Inputs for RSV Decision Tree		
Number of Cases of RSV		
RSV Cases	20.00%	[94]
No RSV Cases	80.00%	Derived
ID™ NOW Accuracy for RSV		
ID™ NOW—RSV Sensitivity	0.99	[89]
ID™ NOW—RSV Specificity	0.98	[89]
ID™ NOW RSV Probability False Negative	0.01	Derived
ID™ NOW RSV Probability False Positive	0.02	Derived
Clinical Judgement Accuracy of RSV		
RSV Clinical Judgement Sensitivity True Positive	0.29	Assumed to be as Influenza
RSV Clinical Judgement Specificity True Negative	0.92	Assumed to be as Influenza
RSV Clinical Judgement Probability False Negative	0.71	Derived
RSV Clinical Judgement Probability False Positive	0.08	Derived
Clinical Consequences of Not Initiating Antiviral Treatment for RSV		
Outcomes for RSV cases without antiviral initiation—Hospitalization	7.60%	[97]
Outcomes for RSV cases without antiviral initiation—Extra Clinic Visit	17.0%	[98]
Outcomes for RSV cases without antiviral initiation—Uncomplicated Cases	0.754	Derived
Clinical Consequences of Initiating Antiviral Treatment for RSV		
Odds ratio of extra outpatient visit with antiviral initiation	0.34	Derived
Odds ratio of hospitalization with antiviral initiation	0.35	Derived
Outcomes for RSV cases with rapid antiviral initiation—Hospitalization	0.0266	Derived
Outcomes for RSV cases with rapid antiviral initiation—Extra Clinic Visit	0.0578	Derived
Outcomes for RSV cases with rapid antiviral initiation—Uncomplicated Cases	0.9156	Derived
RSV ICU Hospitalization Clinical Inputs		
Hospitalization for RSV—ICU admission rate	0.246	[99]
ICU Length of Stay	15 Days	[99]
Mortality (with ICU admission)—RSV	12.80%	[99]
Living Cases (with ICU admission)—RSV	0.872	Derived
RSV Ward Hospitalization Clinical Inputs		
Hospitalization for RSV—Ward admission rate	0.754	Derived
Ward Length of Stay	9 Days	[99]
Mortality (with Ward admission)—RSV	4.60%	[99]
Living Cases (with Ward admission)—RSV	0.934	Derived
RSV Outpatients Clinical Inputs		
Duration of outpatient RSV illness (untreated)	16 Days	[69]
Accelerated factor with early Antiviral Initiation	0.56	Assumed to be as Influenza
Duration of outpatient RSV illness (Treated)	10 Days	Derived

Where RSV: Respiratory Syncytial Virus

Clinical pathways differentiate between untreated cases and those receiving timely antiviral initiation, allowing the model to capture reductions in disease severity, outpatient re-visits, and hospitalization risk.

Hospitalization parameters include ward and ICU admission rates, lengths of stay, and pathogen-specific mortality estimates. For outpatients, the expected duration of illness, treated and untreated, is incorporated (Table 3).

### Treatments

Reflecting Saudi healthcare practice, the model incorporated differentiated treatment pathways for Influenza A&B and RSV across all diagnostic outcomes (true positive, false negative, false positive, and true negative).

For true-positive Influenza A&B cases, patients were assumed to have received Oseltamivir as first-line antiviral therapy in accordance with clinical guidelines. In contrast, true positive RSV cases received supportive management only, consisting primarily of salbutamol nebulization for bronchospasm, as no routine antiviral therapy is recommended for adult RSV infection.

Across both diseases, false negative patients who were hospitalized (ICU or WARD) were modeled as developing complicated lower respiratory tract infection (LRTI). These cases were treated with broad-spectrum antibiotics and systemic corticosteroids and underwent additional diagnostic testing, including viral culture, complete blood count (CBC), and chest X-ray, consistent with standard inpatient practice for severe respiratory infections in geriatric populations. Mortality in both pathways was modeled as 20 ICU days, capturing the full cost of prolonged palliative and supportive care commonly required for older adult patients nearing end-of-life.

For negative cases, treatment depended on diagnostic accuracy. True negative patients received supportive care only, while false positive patients incurred costs of unnecessary therapy—antiviral treatment in the case of Influenza and salbutamol nebulization in the case of RSV.

The model also incorporates the clinical and economic impact of unnecessary antibiotic prescribing among outpatients. Evidence indicates that approximately 42.2% of outpatient encounters for acute respiratory illness receive antibiotics unnecessarily [104]. In this BIA, this probability is applied to patients who do not have influenza or RSV but are incorrectly managed, specifically those in the false-positive and false-negative diagnostic categories. For 80% of these patients [105], the model includes the additional microbial resistance–related cost, reflecting the downstream economic burden associated with inappropriate antibiotic use in both homecare and hospital settings.

### Staff working time per diagnostic

Staff time was incorporated into the model to capture the labor costs associated with sample collection, handling, and diagnostic processing across the three evaluated pathways (mPOCT and viral culture laboratory testing for hospital-based and homecare patients). Salary costs were converted into a cost-per-minute estimate using the standard Saudi working schedule of 22 working days per month and 8 working hours per day.

Nursing time requirements differed by diagnostic pathway. For the mPOCT, the nurse’s active time per sample was estimated at 10 min. For hospital-based laboratory testing, nurse involvement for specimen collection, labeling, transport, preparation, and documentation was estimated at 45 min per sample, while the homecare pathway required a longer interaction time of 75 min per sample due to travel, patient preparation, and on-site and hospital administrative activities. Thus, mPOCT reduces hospital-based laboratory testing time by 77% and homecare-based testing time by 86%.

Pathologist (or clinical microbiologist) involvement was captured only within the diagnostic workflow for laboratory-based testing. The model included 120 min of pathologist working time per processed sample [94, 106].

### Costs and healthcare resource use

All model’s unit costs are presented in Table (S1) in the Appendix. The Budget Impact Analysis included direct medical costs for each diagnostic and clinical management pathway. Cost inputs were derived primarily from the Saudi Price List 2025. All cost parameters were modeled in Saudi Riyals (SAR) for the year 2025 and reflect the perspective of the Saudi healthcare system.

Unit costs included diagnostic procedures (e.g., mPOCT, laboratory viral culture), outpatient and homecare visits, ward and ICU admissions, supportive medications, therapeutic interventions for influenza- or RSV-related complications, and costs for additional diagnostic testing.

Staff-related costs were treated as separate inputs and were calculated based on monthly salaries for nurses and pathologists. These salary inputs were subsequently converted into per-minute labor costs using standard national assumptions for working days and hours. Staff time was then multiplied by diagnostic pathway–specific working minutes to estimate the labor component attributable to each test strategy.

### Sensitivity analysis

A deterministic sensitivity analysis (DSA) was conducted to assess the impact of parameter uncertainty on the robustness of the cost model and to identify the variables exerting the greatest influence on model outcomes. The Delphi parameters were varied by ± 20% from base case. For parameters with base case values expressed as means of multiple estimates, we tested the corresponding minimum and maximum values. Cost parameters, including diagnostic tests, medications, staff salaries, and service utilization, were varied by ± 20% from their base-case values in accordance with standard health-economic practice. Clinical parameters, however, were varied using their reported confidence interval (CI) or standard deviations ranges as published in the referenced clinical studies, ensuring that uncertainty in epidemiological and effectiveness estimates was accurately reflected in the model. The resulting changes in overall budget impact were summarized using Tornado diagrams, allowing clear visualization of the parameters with the strongest influence on cost outcomes and highlighting the relative contribution of clinical versus economic inputs to model uncertainty.

## Base-case results

Base-case results are presented in Table 3 and figures (S1, S2, S3 and S4). Across all modeled pathways—hospital-based and homecare-based older adult patients with Influenza A&B and RSV—the introduction of the mPOCT generated substantial cost reductions compared with current clinical judgement. For Hospital-Based Influenza A&B, total costs decreased from SAR 57.86 million in the current scenario to SAR 26.93 million with mPOCT, resulting in savings of approximately SAR 30.92 million. Similarly, Homecare-Based Influenza A&B costs decreased from SAR 13.25 million to SAR 7.97 million, resulting in savings of SAR 5.28 million. These reductions were primarily driven by the substantial decrease in false-negative cases progressing to complicated LRTI requiring ICU or ward care, as well as by reduced unnecessary antiviral and empirical antibiotic therapy among false positives.

**Table 3:** Base-case results of the budget impact model

Cohort & Infection Type	Current Scenario: Total Cost (SAR)	Future Scenario: Total Cost (SAR)	Difference (SAR)
Hospital-Based Influenza A&B	SAR 57,858,038.91	SAR 26,933,837.90	-SAR 30,924,201.02
Homecare-Based Influenza A&B	SAR 13,254,535.77	SAR 7,974,232.73	-SAR 5,280,303.04
Hospital-Based RSV	SAR 40,640,192.12	SAR 20,706,660.94	-SAR 19,933,531.18
Homecare-Based RSV	SAR 9,157,462.00	SAR 5,988,728.53	-SAR 3,168,733.48
Total Savings	—	—	**-SAR 59,306,768.72**

For RSV, mPOCT also demonstrated strong economic benefit. In the hospital cohort, costs declined from SAR 40.64 million under current practice to SAR 20.71 million with mPOCT—representing savings of SAR 19.93 million. In the homecare cohort, costs decreased from SAR 9.16 million to SAR 5.99 million, resulting in approximately SAR 3.17 million in savings. These results were driven by earlier detection, prevention of severe complications, and reduced escalation from homecare to hospital-based management.

On a per-patient basis per year, adoption of mPOCT generated savings of SAR 609.92 and SAR 694.97 for hospital-based and homecare-based Influenza A&B patients, respectively, and SAR 524.20 and SAR 556.08 for hospital-based and homecare-based RSV patients, respectively.

Overall, the future mPOCT scenario produced total combined savings of SAR 59.31 million across all four decision trees, confirming that mPOCT offers a highly cost-efficient diagnostic strategy for managing respiratory infections in geriatric populations in Saudi Arabia.

Moreover, it was demonstrated that the implementation of mPOCT resulted in substantial AMR-related savings, reducing AMR costs by 53.86% for Influenza A&B and by 57.20% for RSV compared with the total cost of the current clinical judgement, as shown in Tables S2 and S3. The observed reductions in AMR-related costs were driven by improvements in diagnostic accuracy (lower number of false-positive and false-negative cases) with mPOCT, which reduced the number of patients exposed to unnecessary antibiotic treatment compared with clinical judgement alone. Consequently, the downstream AMR-related costs incorporated in the model were substantially reduced.

A scenario analysis was conducted on the risk of AMR of 50% instead of 80% (base case parameter informed by literature). We found that adoption of mPOCT generated savings of SAR 363 and SAR 448 per hospital-based and homecare-based Influenza A&B patient per year, respectively, and SAR 279 and SAR 311 per hospital-based and homecare-based RSV patient per year, respectively.

### Sensitivity-analysis results

**Fig. 3:**
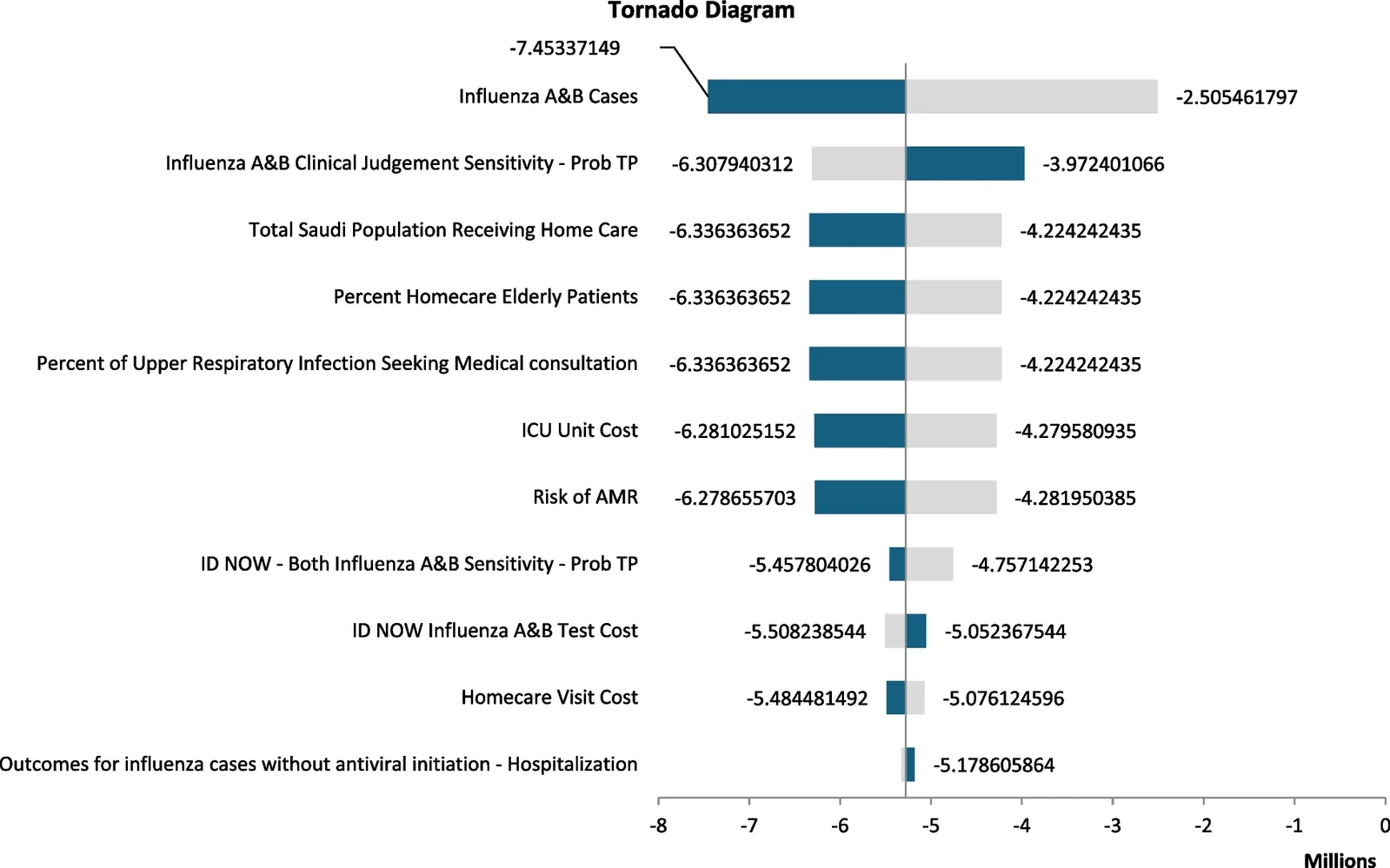
Results of one-way sensitivity analysis for home care patients. The dark colour refers to the higher output value, while the light colour refers to the lower output value

Tornado diagrams show the results of the one-way sensitivity analysis (Figs. 3 & S5). The most impactful parameter is the number of Influenza A&B cases for both home care and hospital-based patients for mPOCT.

## Discussion

Our base-case analysis showed that implementing mPOCT for influenza A&B and RSV in older adult Saudi patients generated substantial savings, under the mPOCT strategy compared with the current clinical-judgement scenario, mainly through reduced hospitalizations, ICU/ward admissions, and unnecessary antivirals and antibiotics. These findings are consistent with international economic evaluations of mPOCT.

In Hong Kong, a study demonstrated that mPOCT for influenza in older adult outpatients was cost-effective compared with clinical judgement alone, reducing hospitalizations and deaths in an ambulatory-care decision tree model [96]. Similarly, in the Netherlands, an economic study reported that introducing mPOCT for influenza/RSV in hospitalized adults reduces hospital costs and improves clinical management compared with standard laboratory testing [107]. In German primary care, the mPOCT for influenza-like illness reduced illness costs for older adult patients by 26% compared to clinical judgement [108]. In the emergency department setting, another economic study demonstrated that an influenza mPOCT strategy was associated with a favourable cost–benefit ratio compared with multiplex RT-PCR, driven by reduced testing costs and more efficient patient flow [109]. Together, these studies further support our conclusion that rapid mPOCT can deliver significant economic and clinical value across diverse healthcare systems.

Based on the International Society for Health Economics and Outcomes Research (ISPOR) Value Framework—which broadens value assessment beyond traditional cost-effectiveness of mPOCT— delivers multidimensional benefits for managing influenza A&B and RSV in Saudi Arabia’s older adult population. Clinically, the platform provides rapid and accurate diagnosis, reducing false negatives, preventing progression to severe lower respiratory tract infections, enabling earlier antiviral initiation, and decreasing inappropriate antibiotic use. These improvements lower ICU and ward admissions and enhance outcomes in high-risk patients. Economically, mPOCT generates meaningful cost offsets by reducing hospitalizations, unnecessary treatments, and diagnostic delays, thereby improving resource allocation during peak respiratory seasons. From a societal perspective, mPOCT reduces diagnostic uncertainty during high-transmission periods, thereby supporting safer, more confident clinical decisions. These ISPOR-defined value dimensions highlight mPOCT as a technology that delivers broad clinical, economic, and societal benefits beyond conventional cost analyses [110].

The mPOCT implementation costs are expected to have a limited impact on the overall budget. Training is typically provided by the manufacturer as part of the device installation process. Similarly, device maintenance costs are not borne by healthcare facilities under current procurement arrangements. According to NUPCO tender requirements, device provision, maintenance, technical support, and replacement of faulty instruments during the warranty period are the responsibility of the supplier. Quality control requirements are also limited. Regarding supply chain costs, logistics and distribution activities are managed through existing procurement mechanisms, with associated handling costs already incorporated within contracted pricing structures. In addition, all materials required to perform the test, including swabs, transfer devices, and controls, are provided as part of the test kit. For these reasons, implementation costs were considered negligible from the health care system perspective.

This study has several notable strengths. It is the first BIA to evaluate the adoption of mPOCT for influenza A&B and RSV specifically within the Saudi older adult population, incorporating both hospital-based and homecare pathways. The model reflects real-world Saudi clinical practice by integrating local epidemiologic data, national cost references, and insights from pre- and post-implementation clinician interviews. Parallel decision trees for influenza and RSV enabled independent assessment of each pathogen’s epidemiology, diagnostic performance, and treatment patterns. The model also accounted for key downstream outcomes—including false negatives, hospital escalation, ICU care, inappropriate antibiotic use and mortality—providing a comprehensive estimate of the clinical and economic consequences of improved diagnostics. Several limitations should be acknowledged. First, as with all modeling studies, results depend on the accuracy of the input parameters; some clinical and epidemiologic estimates were derived from international studies and some assumptions due to limited local data which were validated by our Delphi panel and tested in sensitivity analysis. In addition, treatment-effect estimates were primarily derived from a single published study in older adults due to the limited availability of comparable evidence, while no RSV-specific studies reporting these outcomes were identified; therefore, influenza-based estimates were conservatively applied to the RSV pathway. Second, real-world practice variation across hospitals and home care programs could lead to differences in resource use, treatment decisions, and diagnostic workflows in the model, that was not fully captured. Third, the one-season time horizon reflects the acute nature of influenza and RSV but does not assess longer-term outcomes or multi-season viral variability. Finally, although expert input was instrumental in shaping Saudi-specific practice, the small number of consultations may not capture the full variability across regions and healthcare settings. The AMR-related cost reductions are model-based estimates derived from published evidence and should therefore be interpreted with appropriate caution. Future work drawing on extensive national data would further validate and refine these estimates.

## Conclusion

This BIA demonstrates that implementing the mPOCT for influenza A&B and RSV in Saudi Arabia may provide substantial clinical and economic value, particularly for high-risk older adult populations. By enabling rapid and accurate diagnosis at both hospital and homecare entry points, mPOCT may significantly reduce false negatives, mitigate progression to severe lower respiratory tract infections, and decrease unnecessary antiviral and antibiotic use. These improvements could translate into major reductions in healthcare utilization, including ward and ICU admissions, and may generate considerable cost savings across a single respiratory season.

## Supplementary Information

Supplementary Material 1.

Supplementary Material 2.

Supplementary Material 3.

Supplementary Material 4.

Supplementary Material 5.

Supplementary Material 6.

Supplementary Material 7.

## Data Availability

Included in the paper or Supplementary Information. All data supporting the findings of this study are available within the paper and its Supplementary Information.
