# Supplementary material for: Budget impact analysis of implementing point-of-care molecular testing for influenza A&B and RSV in Saudi older adult patients across hospital and home-care settings
Source: Health Econ Rev. 2026 Oct 7;16:115. doi: 10.1186/s13561-026-00872-3 (PMC13660157; doi:10.1186/s13561-026-00872-3)
Supplement: Supplementary file 1 [file 13561_2026_872_MOESM5_ESM.docx]

| **Figure S1. Budget Impact of Adoption for Hospital-Based Older adult Patients with Influenza A&B in Saudi Arabia** | **Figure S2. Budget Impact of Adoption for Homecare Older adult Patients with Influenza A&B in Saudi Arabia** |
| --- | --- |
|  |  |
| **Figure S3. Budget Impact of Adoption for Hospital-Based Older adult Patients with RSV in Saudi Arabia** | **Figure S4. Budget Impact of Adoption for Homecare Older adult Patients with RSV in Saudi Arabia** |

Budget Impact on Adopting ID NOW for Hospital Based Elderly Patients in KSA - Influenza A&B

|  |  |
| --- | --- |
| Current Scenario Influenza A&B - Clinical Judgement - Hospital Based | 57858039 |
| Future Scenario Influenza A&B - ID NOW - Hospital Based | 26933838 |

Budget Impact on Adopting ID Now for Homecare Elderly Patients in KSA - Influenza A&B

|  |  |
| --- | --- |
| Current Scenario Influenza A&B - Clinical Judgement - Homecare Based | 13254536 |
| Future Scenario Influenza A&B - ID NOW - Homecare Based | 7974233 |

Budget Impact on Adopting ID NOW for Hospital Based Elderly Patients in KSA - RSV

|  |  |
| --- | --- |
| Current Scenario RSV - Clinical Judgement - Hospital Based | 40640192 |
| Future Scenario RSV - ID NOW - Hospital Based | 20706661 |

Budget Impact on Adopting ID NOW for Homecare Elderly Patients in KSA - RSV

|  |  |
| --- | --- |
| Current Scenario RSV - Clinical Judgement - Homecare Based | 9157462 |
| Future Scenario RSV - ID NOW - Homecare Based | 5988729 |

Tornado Diagram

|  |  |  |
| --- | --- | --- |
| Influenza A&B Cases | -14271294 | -43965634 |
| Influenza A&B Clinical Judgement Sensitivity - Prob TP | -37092366 | -23073809 |
| ICU Unit Cost | -24246152 | -37602250 |
| Risk of AMR | -24261964 | -37586438 |
| Elderly Saudi Population Receiving Hospital Care | -24739361 | -37109041 |
| Percent of Upper Respiratory Infection Seeking Medical consultation | -24739361 | -37109041 |
| ID NOW - Both Influenza A&B Sensitivity - Prob TP | -27784044 | -31989611 |
| ID NOW Influenza A&B Test Cost | -32445267 | -29403135 |
| Hospital Based Visit Cost | -30413252 | -31435150 |
| Outcomes for influenza cases without antiviral initiation - Hospitalization | -31238076 | -30245552 |
| Antibiotics Prescribed Unnecessarily for Outpatients | -30529457 | -31239997 |

**Figure S5: Results of one-way sensitivity analysis for hospital-based patients**

**The dark colour refers to the higher output value, while the light colour refers to the lower output value**
