# Supplementary material for: Budget impact analysis of implementing point-of-care molecular testing for influenza A&B and RSV in Saudi older adult patients across hospital and home-care settings
Source: Health Econ Rev. 2026 Oct 7;16:115. doi: 10.1186/s13561-026-00872-3 (PMC13660157; doi:10.1186/s13561-026-00872-3)
Supplement: Supplementary file 2 [file 13561_2026_872_MOESM6_ESM.docx]

1. **Calculations:**

| **Derived Clinical Input** | **Calculation Method** |  | **Derived Clinical Input** | **Calculation Method** |
| --- | --- | --- | --- | --- |
| No Influenza A&B Cases | Calculated as 100% minus the proportion of Influenza A&B cases. |  | No RSV Cases | Calculated as 100% minus the proportion of RSV cases. |
| ID NOW Influenza A&B Probability False Negative | Calculated as 100% minus the sensitivity of ID NOW for Influenza A&B. |  | ID NOW RSV Probability False Negative | Calculated as 100% minus the sensitivity of ID NOW for RSV. |
| ID NOW Influenza A&B Probability False Positive | Calculated as 100% minus the specificity of ID NOW for Influenza A&B. |  | ID NOW RSV Probability False Positive | Calculated as 100% minus the specificity of ID NOW for RSV. |
| Influenza A&B Clinical Judgement Probability False Negative | Calculated as 100% minus the sensitivity of clinical judgement for Influenza A&B. |  | RSV Clinical Judgement Probability False Negative | Calculated as 100% minus the sensitivity of clinical judgement for RSV. |
| Influenza A&B Clinical Judgement Probability False Positive | Calculated as 100% minus the specificity of clinical judgement for Influenza A&B. |  | RSV Clinical Judgement Probability False Positive | Calculated as 100% minus the specificity of clinical judgement for RSV. |
| Outcomes for Influenza Cases without Antiviral Initiation – Uncomplicated Cases | Calculated as the remaining proportion after accounting for hospitalization and extra clinic visits. |  | Outcomes for RSV Cases without Treatment Initiation – Uncomplicated Cases | Calculated as the remaining proportion after accounting for hospitalization and extra clinic visits. |
| Outcomes for Influenza Cases with Rapid Antiviral Initiation – Hospitalization | Calculated by applying the odds ratio of hospitalization with antiviral initiation to the hospitalization probability without antiviral treatment. |  | Outcomes for RSV Cases with Rapid Management – Hospitalization | Calculated by applying the odds ratio of hospitalization associated with early management to the hospitalization probability without early management. |
| Outcomes for Influenza Cases with Rapid Antiviral Initiation – Extra Clinic Visit | Calculated by applying the odds ratio of extra outpatient visits with antiviral initiation to the probability of extra clinic visits without antiviral treatment. |  | Outcomes for RSV Cases with Rapid Management – Extra Clinic Visit | Calculated by applying the odds ratio of extra outpatient visits associated with early management to the probability of extra clinic visits without early management. |
| Outcomes for Influenza Cases with Rapid Antiviral Initiation – Uncomplicated Cases | Calculated as the remaining proportion after accounting for hospitalization and extra clinic visits following antiviral initiation. |  | Outcomes for RSV Cases with Rapid Management – Uncomplicated Cases | Calculated as the remaining proportion after accounting for hospitalization and extra clinic visits following early management. |
| Hospitalization for Influenza – Ward Admission Rate | Calculated as the remaining proportion of hospitalized patients after accounting for ICU admissions. |  | Hospitalization for RSV – Ward Admission Rate | Calculated as the remaining proportion of hospitalized patients after accounting for ICU admissions. |
| Living Cases (with ICU Admission) – Influenza | Calculated as the remaining proportion of patients after accounting for ICU mortality. |  | Living Cases (with ICU Admission) – RSV | Calculated as the remaining proportion of patients after accounting for ICU mortality. |
| Living Cases (with Ward Admission) – Influenza | Calculated as the remaining proportion of patients after accounting for ward mortality. |  | Living Cases (with Ward Admission) – RSV | Calculated as the remaining proportion of patients after accounting for ward mortality. |
| Duration of Outpatient Influenza Illness (Treated) | Calculated by applying the accelerated recovery factor to the duration of untreated influenza illness. |  | Duration of Outpatient RSV Illness (Treated) | Calculated by applying the accelerated recovery factor to the duration of untreated RSV illness. |
