# Supplementary material for: Budget impact analysis of implementing point-of-care molecular testing for influenza A&B and RSV in Saudi older adult patients across hospital and home-care settings
Source: Health Econ Rev. 2026 Oct 7;16:115. doi: 10.1186/s13561-026-00872-3 (PMC13660157; doi:10.1186/s13561-026-00872-3)
Supplement: Supplementary file 3 [file 13561_2026_872_MOESM7_ESM.docx]

**Table (S1): Model Unit Costs**

| **Input** | **Base-Case** | **Lower-Case** | **Upper-Case** | **Reference** |
| --- | --- | --- | --- | --- |
| **Unit Costs** | | | | |
| Influenza A&B Test Cost | SAR 150.00 | 120 | 180 | Company Price |
| Hospital Based Visit Cost | SAR 300.00 | 240 | 360 | Saudi Price List 2025 |
| Homecare Visit Cost | SAR 800.00 | 640 | 960 | Saudi Price List 2025 |
| Antiviral "Oseltamivir" Cost - Bottle | SAR 98.30 | 78.64 | 117.96 | Saudi Price List 2025 |
| ICU Unit Cost | SAR 1,500.00 | 1200 | 1800 | Saudi Price List 2025 |
| Ward Unit Cost | SAR 800.00 | 640 | 960 | Saudi Price List 2025 |
| Viral Lab Tests for False Negative | SAR 260.00 | 208 | 312 | Saudi Price List 2025 |
| Symbicort® 160/4.5 mcg (budesonide/formoterol) | SAR 134.00 | 107.2 | 160.8 | Saudi Price List 2025 |
| Solu-Pred 1 g Injection | SAR 72.00 | 57.6 | 86.4 | Saudi Price List 2025 |
| Piperacillin–Tazobactam 4 g / 0.5 g (4.5 g) Vial | SAR 15.35 | 12.28 | 18.42 | Saudi Price List 2025 |
| Chest X-ray | SAR 150.00 | 120 | 180 | Saudi Price List 2025 |
| CBC | SAR 150.00 | 120 | 180 | Saudi Price List 2025 |
| Empiric Antibiotic - Amoxicillin–Clavulanate 875/125 mg | SAR 0.40 | 0.32 | 0.48 | Saudi Price List 2025 |
| Salbutamol | SAR 2.63 | 2.104 | 3.156 | Saudi Price List 2025 |
| Panadol 500mg | SAR 0.05 | 0.04 | 0.06 | Saudi Price List 2025 |
| Antihistamine Tablet | SAR 0.12 | 0.096 | 0.144 | Saudi Price List 2025 |
| **Staff Working Costs** |  |  |  |  |
| Nurse Salary Per Month | SAR 6,000.00 | 4800 | 7200 | [109] |
| Pathologist Salary Per Month | SAR 24,583.33 | 19,666.67 | 29,500.00 | [110] |

**Table (S2): AMR-related savings in Influenza A&B**

| **Antimicrobial Resistance Costs – Influenza A&B** | | | |
| --- | --- | --- | --- |
| Total Cost of Antimicrobial Resistance Hospital Based - Influenza - Clinical Judgement | SAR 34,376,367.42 | Total Cost of Antimicrobial Resistance Hospital Based - Influenza - | SAR 1,065,183.22 |
| Total Cost of Antimicrobial Resistance Homecare Based - Influenza - Clinical Judgement | SAR 5,151,383.63 | Total Cost of Antimicrobial Resistance Homecare Based - Influenza - | SAR 159,620.34 |
| Total AMR Hospital and Homecare Influenza - Clinical Judgement | SAR 39,527,751.05 | Total AMR Hospital and Homecare Influenza - | SAR 1,224,803.55 |
| **Total Antimicrobial Saving for Influenza A&B** | | | **-SAR 38,302,947.50** |
| **Percent AMR Saving = ((AMR Cost_Current – AMR Cost_) / Total Cost_Current) × 100** | | | **53.86%** |

**Table (S3): AMR-related savings in RSV**

| **Antimicrobial Resistance Costs – RSV** | | | |
| --- | --- | --- | --- |
| Total Cost of Antimicrobiaal Resistance Hospital Based - RSV - Clinical Judgement | SAR 25,271,308.83 | Total Cost of Antimicrobiaal Resistance Hospital Based - RSV - | SAR 498,307.50 |
| Total Cost of Antimicrobiaal Resistance Homecare Based - RSV - Clinical Judgement | SAR 3,786,968.10 | Total Cost of Antimicrobiaal Resistance Homecare Based - RSV - | SAR 74,672.61 |
| Total AMR Hospital and Homecare RSV - Clinical Judgement | SAR 29,058,276.93 | Total AMR Hospital and Homecare RSV - | SAR 572,980.11 |
| **Total Antimicrobial Saving for Influenza A&B** | | | **-SAR 28,485,296.82** |
| **Percent AMR Saving = ((AMR Cost_Current – AMR Cost_) / Total Cost_Current) × 100** | | | **57.20%** |
